# Mechanics of amorphous solids—identification and constitutive modelling

**DOI:** 10.1007/s11043-018-9384-1

**Published:** 2018-04-06

**Authors:** J. A. W. van Dommelen, R. Estevez

**Affiliations:** 1 Department of Mechanical Engineering, Eindhoven University of Technology, Eindhoven, The Netherlands; 2 SIMAP, Materials Science Laboratory, University of Grenoble, Grenoble, France

Both polymers and metals can be in an organised crystalline or amorphous glassy state, where for polymers usually at least a part of the structure is amorphous and metals are in a glassy state only when processed under special conditions. At the *15th European Mechanics of Materials Conference* in September 2016 in Brussels, Belgium, a session focussing on the mechanical properties of amorphous or partly amorphous solid materials was organised, attempting to bridge descriptions found for metallic glasses and polymers, which share some common features, such as a rate- and temperature-dependent response, being prone to strain localisation in the form of shear bands, the occurrence of damage by cavitation, etc.

This lively and fruitful session hosted contributions on new developments in the mechanics of polymers and amorphous metals and superalloys. Among the common themes for these various materials were mechanisms and activation of plasticity and plastic localisation, as well as the rate dependence of these materials, including resonance and vibration. The session included contributions on failure mechanisms of both polymers and metals in the form of fatigue, embrittlement & damage and toughening. Methods for testing and identification were presented, which are of key importance for characterisation of the time-dependent behaviour of solids. A part of the session was specifically dedicated to physically-based constitutive modelling and fatigue prediction of elastomers and rubber-like materials. Finally, the session included several papers on polymerisation, structure formation and micro-patterned materials.

Based on the inspiring session, this special issue on the mechanics of amorphous solids has been assembled, with all contributed papers from presenters at the *15th European Mechanics of Materials Conference*. While being a European conference, it contains the results of research work from several continents. Besides Europe, contributions originate from the USA, Russia, India, Algeria and Taiwan.

A common theme within this special issue is the constitutive behaviour of various amorphous materials. In particular, the physical origin and localisation of plastic deformation are addressed, including their temperature and rate dependence. A new hyperelastic model for rubber-like materials is proposed, focussing on their multi-axial behaviour. Next, the effect of holes, cracks and inclusions in anisotropic viscoelastic solids is theoretically and computationally investigated using the elastic-viscoplastic correspondence principle with time-invariant boundaries. An experimental characterisation method for non-linear viscoelastic materials is presented, accounting for the cyclic response under quasi-static pre-tension, where both the quasi-static and large strain-rate response is obtained. For identification of the material parameters in visco-elasticity from constant strain-rate, relaxation, and creep tests, an approach based on Bayesian interference is presented. For identification of material parameters, a theoretical model of the friction-induced localised plastic deformation in axial friction tests with viscoplastic materials is presented. Finally, the results of a computational study on the crimping and expansion of bioresorbable polymeric stents are shown.

The comprehensive but coherent range of topics of this special issue elucidates the importance of the mechanics of amorphous solids, where many aspects still need to be resolved. In this, researchers working on metals and polymers face similar challenges and can benefit from close interaction with each other. Therefore, at the *16th European Mechanics of Materials Conference* in 2018 in Nantes, France, again a session focussing on the mechanical properties of amorphous solid materials will be organised.

